# Structural and Biological Behaviour of Co(II), Cu(II) and Ni(II) Metal Complexes of Some Amino Acid Derived Schiff-Bases

**DOI:** 10.1155/MBD.1997.267

**Published:** 1997

**Authors:** Zahid H. Chohan, M. Praveen, A. Ghaffar

**Affiliations:** 1 Department of Chemistry University of Aberdeen Scotland Old Aberdeen AB9 2UE UK; 2 Department of Chemistry Islamia University Bahawalpur Pakistan

## Abstract

Biologically active tridentate amino acid (Alanine, Glycine & Tyrosine) derived Schiff-bases and their Co(II), Cu(II) & Ni(II) complexes have been synthesised and characterised on the basis of their conductance and magnetic measurements, elemental analysis and ^13^C-NMR, ^1^H-NMR, IR and electronic spectral data. These Schiff-bases and their complexes have been evaluated for their antibacterial activity against bacterial species such as *Staphylococcus aureus, Escherichia coli, Klebsiella pneumonae, Proteus vulgarus* and *Pseudomonas aeruginosa* and this activity data show the metal complexes to be more antibacterial than the Schiff-bases against one or more bacterial species.


### STRUCTURAL AND BIOLOGICAL BEHAVIOUR OF Co(II), Cu(II) AND Ni(II) METAL COMPLEXES OF SOME AMINO ACID DERIVED SCHIFF-BASES

Zahid . H. Chohan*[1], M. Praveen[1] and A. Ghaffar[2]

[1] Department of Chemistry, University of Aberdeen, Old Aberdeen, AB9 2UE, Scotland, U.K
[2] Department of Chemistry, Islamia University, Bahawalpur, Pakistan

**Abstract**
Biologically active tridentate amino acid (Alanine, Glycine & Tyrosine) derived Schiff-bases and their Co(II), Cu(II) & Ni(II) complexes have been synthesised and characterised on the basis of their conductance and magnetic measurements, elemental analysis and $^{13}$C-NMR, $^{1}$H-NMR, IR and electronic spectral data. These Schiff-bases and their complexes have been evaluated for their antibacterial activity against bacterial species such as *Staphylococcus aureus, Escherichia coli, Klebsiella pneumonae, Proteus vulgarus* and *Pseudomonas aeruginosa* and this activity data show the metal complexes to be more antibacterial than the Schiff-bases against one or more bacterial species.

### Introduction
Transition metal chemistry of virtually all naturally occuring amino acids has been studied in details[1-4]. Almost all amino acids have been found to coordinate solely through the amino and carboxylato groups forming a stable five membered chelate ring system with the metal ion [5,6]. In the present studies we have introduced an azomethine linkage which may permit a variety in their coordination behaviour and complexation role.Therefore, novel Schiff-bases $HL_1$, $HL_2$ and $HL_3$ (Fig 1) were synthesised by the reaction of amino acids e. g; tyrosine, alanine & glycine with indole-3-carboxaldehyde and their co-ordination behaviour with Co(II), Cu(II) and Ni(II) metal ions was investigated.

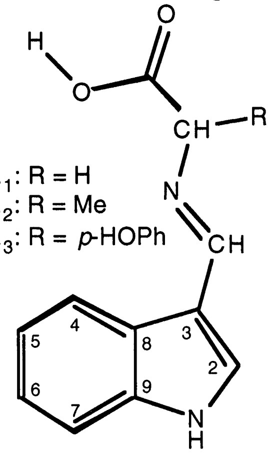

Fig 1 Structure of Schiff-bases

### Experimental
### Material and Methods
All chemicals and solvents used were of Analar grade. All the metals were used as their chloride salts. Infrared and UV spectra were recorded on IR 408 - Shimadzu and Hitachi U-2000 double-beam Spectrophotometers. Conductance of the metal complexes was determined in DMF on a YSI-32 model conductometer. Melting points were recorded on Gallenkamp apparatus and are uncorrected. $^{1}$H-NMR and $^{13}$C-NMR spectra of Schiff-bases in DMSO-$d_6$ were recorded on a $R_{10}$ and Brucker 250 MHz Spectometer.

* Present address : Department of chemistry, Meston Walk, University of Aberdeen, Old Aberdeen AB9 2UE, Scotland (U.K)

Elemental analysis was carried out on a Coleman automatic analyser. Magnetic measurements were done on solid complexes using the Gouy method. The antibacterial studies were carried out with the help of the Department of Pathology, Quaid-e-Azam Medical College, Bahawalpur (Pakistan).

In view of the promising physiological properties of various amino acids and their derivatives, we thought it of interest to study the biological role of metal ions on these synthesised amino acid derived Schiff-bases. This role of metal ions on the biological properties was studied against bacterial species such as *Staphylococcus aureus, Escherichia coli, Klebsiella pneumonae, Proteus vulgarus* and *Pseudomonas aeruginosa.*

**Preparation of Schiff-bases**

To an ethanolic solution (20 ml) of the respective amino acid (0. 01 M) were added indole-3-carboxaldehyde (0. 01 M) in ethanol (10 ml) with stirring. Then 2-3 drops of conc. $H_2SO_4$ were added and this mixture was refluxed for 1h. The reaction mixture was then cooled in an ice-bath which immediately gave a precipitated product. The product thus obtained was filtered, washed with ethanol (1 x 5 ml) then with ether (2 x 5 ml) and dried. The crude product was crystallised from aqueous ethanol to give $HL_1$ (70%), $HL_2$ (68%) and $HL_3$ (65%.)

**Preparation of Metal Complexes**

To a hot ethanolic solution (20 ml) of the Schiff-base (0. 02 M) was added an aqueous solution (10 ml) of the respective metal (II) chloride (0. 01 M) salt. The mixture was refluxed for 2 h. The resulting mixture was cooled, filtered and reduced nearly half its volume. This concentrated solution was left overnight at room temperature which resulted in the formation of a solid product. The product thus obtained was filtered, washed with ethanol (1x10 ml) and dried. Crystallisation from aqueous ethanol gave **1** (69%), **2** (65%), **3** (67%), **4** (67%), **5** (65%), **6** (66%), **7** (70%), **8** (65%) and **9** (68%).

**Antibacterial Studies**

The synthesised metal complexes and the free Schiff bases were screened for their antibacterial activity against bacterial species *Staphylococcus aureus (a), Escherichia coli (b), Klebsiella pneumonae (c), Proteus vulgarus (d)* and *Pseudomonas aeruginosa (e).* The paper disc diffusion method[23-25] was used for the determination of antibacterial activity

**Preparation of Discs**

A Schiff base / complex (30μg) in DMF (0. 01 ml) was applied on a paper disc prepared from blotting paper (3 mm diameter) with the help of a micropipette. The discs were left in an incubator for 48 h at 37°C and then applied on bacteria grown agar plates

**Preparation of Agar Plates**

Minimal agar was used for the growth of specific bacterial species. For the preparation of agar plates for *Escherichia coli*, MacConkey agar (50 g), obtained from Merck Chemical Company, was suspended in freshly distilled water (1 L). It was allowed to soak for 15 minutes and then boiled on a water bath until the agar was completely dissolved. The mixture was autoclaved for 15 minutes at 120_ C and then poured into previously washed and sterilised petri dishes and stored at 40° C for inoculation.

**Procedure of Inoculation**

Inoculation was done with the help of a platinum wire loop which was made red hot on a flame, cooled and then used for the application of bacterial strains.

**Application of Discs**

A sterilised forcep was used for the application of paper disc on the already inoculated agar plates. When the discs were applied, they were incubated at 37° C for 24 h. The zone of inhibition was then measured (in diameter) around the disc.

**Results and discussion**

Schiff-bases were prepared by the same method as reported earlier[7-9].The structural determination of synthesised Schiff bases $HL_1$, $HL_2$ and $HL_3$ was done on the basis of their IR, $^1$H-NMR, $^{13}$C-NMR and CHN analytical data (Table 1 & 2).

The IR spectra of the Schiff bases (Table 1) show characteristic absorption bands at 3465, 3380, 1730, 1625 and 1545 $cm^{-1}$ due to (-OH), (-NH), (-C = O), (-C = N) and (C = C) stretching vibrations respectively. The appearance of the new bands at 1625 $cm^{-1}$ due to the Schiff-base azomethine linkage confirm the formation of the ($HL_1$, $HL_2$ and $HL_3$). The $^1$H-NMR and $^{13}$C-NMR spectral data (Table 2) was assigned by comparing the chemical shifts of these molecules to those of the known[10] similar structures. The CHN analytical data (Table 1) also is found to be in agreement with the expected molecular structures of the Schiff bases and their metal complexes (Table 3).

**Table 1** **Physical, Spectral and Analytical Data of the Schiff bases**

| Schiff-base/ Mol.Form | M.P. (°C) | IR ($cm^{-1}$) | Cal(Found)% C | H | N |
|---|---|---|---|---|---|
| **$HL_1$** $C_{11}H_{10}N_2O_2$ | 180-182 | 3465,3382,2915, 2865,2015,1945, 1730,1625,1545, 1350,875 | 65.36 (65.93) | 4.94 (4.87) | 13.85 (13.69) |
| **$HL_2$** $C_{12}H_{11}N_2O_2$ | 142-143 | 3465,3380,2965, 2015,1945,1730,169[illegible] ,1625,1545, 1410,1055,875 | 66.99 (67.62) | 5.11 (4.29) | 13.01 (12.78) |
| **$HL_3$** $C_{17}H_{13}N_2O_3$ | 168-170 | 3465,3380,2915, 2860,2115,2025, 1940,1730,1695, 1625,1545,1355, 1295,1110,1055, 875 | 69.64 (70.08) | 4.43 (3.86) | 9.52 (10.16) |

**Table 2** **$^1$H-NMR and $^{13}$C-NMR Data of the Schiff bases**

| Schiff-base | $^1$H-NMR (DMSO-$d_6$) (ppm) | $^{13}$C-NMR (DMSO-$d_6$) (ppm) |
|---|---|---|
| **$HL_1$** | 3.87(s,2H,NC*H*$_2$),4.68(s,1H),6.13(s,1H, azomethine),7.46-7.48(m,2H),7.91-7.94 (m,2H),8.33(s,1H,N*H*),8.83(s,1H,O*H*) | 61.32(NCH$_2$),104.71($C_3$),112.24 ($C_7$),121.36($C_5$),123.22($C_4$),124.12 ($C_6$),126.20($C_2$),129.12($C_8$),137.27 ($C_9$),152.31(*C*=N),187.38(*C*=O) |
| **$HL_2$** | 1.87(s,3H,C*H$_3$*),3.48(s,NC*H*),4.62(s,1H), 6.15(s,1H,azomethine),7.44-7.46(m,2H), 7.92-7.94(m,2H),8.34(s,1H,N*H*),8.85(s, 1H,O*H*) | 27.4(*C*H$_3$),60.23(NCH),104.6(C3), 112.21($C_7$),121.18($C_5$),123.21($C_4$), 124.57($C_6$),126.08($C_2$),129.50($C_8$), 137.46($C_9$),153.11(*C*=N),186.92 (*C*=O) |
| **$HL_3$** | 3.81(s,1H,NC*H*),4.66(s,1H),6.12(s,1H, azomethine),7.43-7.45(m,2H),7.51-7.52 (m,2H),7.89-7.91(m,2H),8.218.23(m,2H), 8.36(s,1H,N*H*),8.76(s,1H),8.87(s,1H,O*H*) | 70.27(NCH-Ph),104.11($C_3$),112.56 ($C_7$),117.18(Ph),121.31($C_5$),121.5(Ph) ,123.46($C_4$),124.41($C_6$),126.82($C_2$), 129.38($C_8$),133.11(Ph),137.22 ($C_9$),153.0(*C*=N),157.18(*C*-OH), 188.27(*C*=O) |

The metal complexes (1-9) of the title Schiff bases were prepared by reacting the metal(II) chloride and the Schiff base in the molar ratio M:L = 1:2. All the complexes are stable crystalline solids which decompose without melting. The conductivity behaviour of these complexes in DMF show their low conductivity values (14-18 $ohm^{-1} cm^2 mol^{-1}$) observed for these complexes in DMF suggest[11, 12] them to be non-electrolyte.

The magnetic susceptibility data (Table 3) of all the solid complexes at room temperature indicate three unpaired electrons for Co(II) ion ($\mu_{eff}$ = 3.6 - 3.65 B.M), one unpaired electron for Cu(II) ion ($\mu_{eff}$ = 1.82 - 1.87 B.M) and two unpaired electrons per Ni(II) ion ($\mu_{eff}$ = 3.12 - 3.15 B.M) suggesting[13 - 15] an octahedral geometry for Co(II) and Ni(II) complexes and a distorted octahedral geometry for Cu (II) complexes.

The bonding of the ligand to the metal ion was investigated by comparing the infrared spectra of the free Schiff bases with their metal complexes (Table 3) indicating that the ligands are coordinated to the metal atom in three ways, thus, representing all the ligands to act as tridentates. It indicated that $\upsilon$ (-C = N) band in the spectra of the Schiff bases at 1625 $cm^{-1}$ due to azomethine linkage was shifted towards lower frequency (5-10 $cm^{-1}$), respectively, indicating that the ligands are coordinated to the metal atoms through azomethine nitrogen. Also, in the spectra of the Schiff bases the bands due to $\nu$ (C = O) at 1730 $cm^{-1}$ shifted towards lower frequency side and the band due to $\nu$ (OH) at 3465 $cm^{-1}$ disappeared which was an evidence for the coordination of these groups with the metal atom. However, the band at 3380 $cm^{-1}$ due to $\nu$ (-NH) of indole remained unchanged indicating that it is not involved in the coordination. Moreover, the new bands appearing in the spectra of the metal complexes and not observed in the spectra of the free Schiff bases within 445 - 450 $cm^{-1}$ and 435-440 $cm^{-1}$ assigned[16, 17] to M - O and M - N modes, conclusively indicated that the ligands are coordinated to the metal ions through these groups.

**Table 3** **Physical Spectral and Analytical Data of the Complexes**

| Complex/ M.Formula | M.P °C Dec. | B.M. ($\mu_{eff}$) | IR ($cm^{-1}$) | $\lambda_{max}$($cm^{-1}$) | Calc (Found)% C H N |
|---|---|---|---|---|---|
| 1 $[Co(L_1)_2Cl_2]$ $C_{22}H_{18}Cl_2CoN_4O_4$ | 247-249 | 3.65 | 3380,3025, 2965,2015,1945, 1810,1723,1615, 1547,1410,1052, 875,450,435 | 30250,17945, 8950 | 49.66 3.38 10.53 (50.46)(2.86)(10.43) |
| 2 $[Co(L_2)_2Cl_2)]$ $C_{24}H_{20}Cl_2CoN_4O_4$ | 240-242 | 3.62 | 3380,2915, 2865,2015,1880, 1722,1621,1544, 1412,1054,875, 510,450 | 29100,17550, 8250 | 51.64 3.58 10.03 (52.60)(3.86)(9.27) |
| 3 $[Co(L_3)_2Cl_2]$ $C_{34}H_{24}Cl_2CoN_4O_6$ | 225-227 | 3.60 | 3380,3165, 2915,2860,2025, 2115,1940,1720, 1622,1547,1295, 875,451,440 | 31000,18215, 9850 | 57.18 3.36 7.84 (57.08)(2.18)(8.62) |
| 4 $[Cu(L_1)_2Cl_2]$ $C_{22}H_{18}Cl_2CuN_4O_4$ | 247-249 | 1.87 | 3380,3025, 2965,2015,1945, 1721,1620,1545, 1412,1351,875, 445,435 | 30795,22950, 13170 | 49.23 3.35 10.43 (49.46)(3.68)(11.12) |
| 5 $[Cu(L_2)_2Cl_2]$ $C_{24}H_{20}Cl_2CuN_4O_4$ | 256-258 | 1.82 | 3380,2915, 2865,2015,1945, 1880,1720,1615, 1544,1412,1054, 875,445,438 | 30270,22550, 12500 | 51.22 3.55 9.95 (51.50)(3.76)(9.62) |
| 6 $[Cu(L_3)_2Cl_2]$ $C_{34}H_{24}Cl_2CuN_4O_4$ | 215-217 | 1.85 | 3380,3165, 2915,2860,2115, 1940,1722,1695, 1615,1545,1295, 1110,875,445,440 | 31500,23170, 13750 | 56.81 3.33 7.70 (55.98)(3.61)(7.52) |
| 7 $[Ni(L_1)_2Cl_2]$ $C_{22}H_{18}Cl_2NiN_4O_4$ | 235-237 | 3.15 | 3380,3025, 2965,2015,1945, 1810,1720,1695, 1620,1545,1410, 1055,875,445,440 | 28350,16915, 9745 | 49.68 3.38 10.52 (49.56)(3.25)(10.14) |
| 8 $[Ni(L_2)_2Cl_2]$ $C_{24}H_{20}Cl_2NiN_4O_6$ | 250-252 | 3.12 | 3380,2915, 2860,2015,1945, 1722,1690,1615, 1544,1412,1054, 875,515,445 | 28500,16250, 9555 | 51.67 3.59 10.03 (51.58)(2.12)(10.17) |
| 9 $[Ni(L_3)_2Cl_2]$ $C_{34}H_{24}Cl_2NiN_4O_6$ | 210-212 | 3.15 | 3380,3165, 2915,2860,2025, 1940,1720,1695, 1620,1545,1295, 1110,1055,875, 445,435 | 28950,17500, 10220 | 57.19 3.36 7.84 (57.12)(3.28)(7.69) |

The electronic spectra of the metal complexes (Table 3) show different $\lambda_{max}$ values with various metal(II) ions which is due to the perturbing influence of the central metal atom on the chelate group. The shift of the band with the change of different colours of the complexes however, strengthen the evidence of complex formation. In these spectra, the Co(II) complexes exhibit bands between 29100 - 3100, 17550 - 18215 and 8250 - 9850 cm-

[1] region assigned to the transitions $^4T_{1g}(F)$ (P) $^4T_{2g}(F)(V_1)$, $^4T_{1g}(F) \rightarrow {}^4A_{2g}(F)(V_2)$ and $^4T_{1g}(F) \rightarrow {}^4T_{1g}(P)(V_3)$ in an octahedral geometry[18, 19]. The spectra of Cu(II) complexes exhibit three broad bands in the region 30270 - 31500, 22550 - 23170 and 12500 - 13750 $cm^{-1}$. The lower energy band may be assigned as 10 Dq band for a distorted octahedral configuration[20, 21] corresponding to the transition $^2E_g \rightarrow {}^2T_{2g}$. The second band can be attributed to intra - ligand charge transfer and the highest energy band is assigned to charge transfer transitions. The electronic spectra of Ni (II) complexes showed three bands between 28350 - 28950, 16250 - 17500 and 9555 - 10220 $cm^{-1}$ region assignable, respectively, to the transitions $^3A_{2g}(F) \rightarrow {}^3T_{2g}(F)(V1)$, $^3A_{2g}(F) \rightarrow {}^3T_{1g}(F)(V2)$ and $^3A_{2g}(F) \rightarrow {}^3T_{2g}(P)(V3)$ suggest their of their octahedral geometry[22, 23].

On the basis of the above observations it is, therefore, proposed that all the complexes show octahedral geometry in which the two ligands behaving as tridentate possibly accommodate themselves around the metal atom in such a way that a stable configuration of a chelate ring is formed (Figure 2).

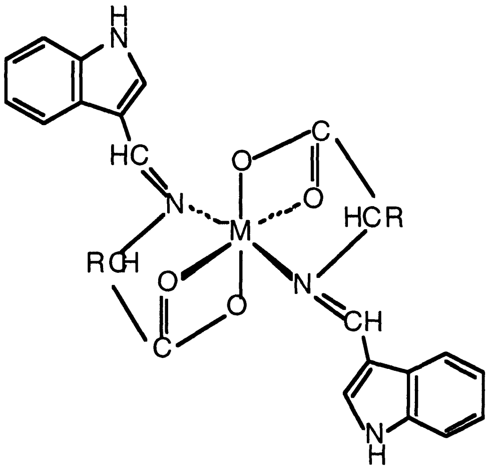

Figure 2 Proposed structure for the metal complex

**Table 4. Antibacterial Activity Data**

| Schiffbases/ Complexes | Microbial Species a | b | c | d | e |
|---|---|---|---|---|---|
| **HL$_1$** | ++ | ++ | + | ++ | + |
| **HL$_2$** | + | ++ | ++ | + | + |
| **HL$_3$** | ++ | + | - | + | + |
| **1** | +++ | +++ | ++ | ++ | ++ |
| **2** | +++ | +++ | ++ | +++ | +++ |
| **3** | ++ | +++ | ++ | ++ | ++ |
| **4** | +++ | ++ | +++ | ++ | +++ |
| **5** | ++ | ++ | ++ | +++ | ++ |
| **6** | +++ | +++ | ++ | ++ | ++ |
| **7** | +++ | ++ | +++ | +++ | ++ |
| **8** | ++ | +++ | ++ | +++ | + |
| **9** | +++ | ++++ | +++ | ++ | ++ |

a = *Staphylococcus aureus*, b = *Escherichia coli*, c = *Klebsiellapneumonae*
d = *Proteus vulgarus*, e = *Pseudomonas aeruginosa*
Inhibition zone diameter (mm), + : 6-10; ++ : 10-14; +++ : 14-18; ++++ : 18-22

**Antibacterial Studies**

The title Schiff bases $HL_1$, $HL_2$ and $HL_3$ and their metal complexes 1-9 were tested for their antibacterial properties against *Staphylococcus aureus (a), Escherichia coli (b), Klebsiella pneumonae (c), Proteus vulgarus (d)* and *Pseudomonas aeruginosa (e)*. All the compounds were evaluated at a concentration of 30 µg / 0. 01 ml in DMF solution using the paper disc diffusion method[24 - 26].
These studies (Table 4) show that all the free Schiff bases are biologically active and their metal complexes show more significant activity against one or more bacterial species than the uncomplexed free Schiff bases.

**Acknowledgement**
We are highly grateful to the Department of Pathology, Quaid-e-Azam Medical College, Bahawalpur, for their kind help in undertaking the antibacterial studies.

**References**

1. H. C. Freeman, Adv. Protein. Chem., 1967, **22**, 257
2. M. N. Hughes, " The Inorganic Chemistry of Biological Process ", John Wiley, New York, 1972, p. 63.
3. S. T. Chow and C. A. McAuliffe, Prog. Inorg. Chem., 1975, **19**, 51
4. T. R. Rao, M. Sahay and R. C. Aggarwal, Ind. J. Chem., 1984, **23 (A)**, 214
5. A. Gergly, I. SoVago, I. Nagypal and R. Kiraly, Inorg. Chim. Acta., 1972, **6**, 435
6. N. Vishnumurthy and P. Lingaiah, Ind. J. Chem., 1986, **25 (A)**, 875
7. Z. H. Chohan and H. Pervez, Synth. React. Inorg. Met.-Org. Chem., 1996, **23**, 1061
8. Z. H. Chohan and S. Kausar, Chem. Pharm. Bull., 1993, **41**, 951
9. Z. H. Chohan and S. Kausar, Chem. Pharm. Bull., 1992, **40**, 2555
10. D. H. Williams and I. Fleming, " Spectroscopic Methods in Organic Chemistry ", 4th Ed, Mc Graw Hill, London, 1989
11. A. M. Shallary, M. M. Moustafa and M. M. Bekheit., J. Inorg. Nucl. Chem., 1979, **41**, 267
12. W. J. Geary, Coord. Chem. Rev., 1971, **7**, 81
13. M. D. Glick and R. L. Lintvedt, Prog. Inorg. Chem., 1976, **21**, 233
14. C. J. Balhausen, " An Introduction to Ligand Field ", Mc Graw Hill, New York, 1962, p. 256,
15. A. B. P. Lever, Inorg. Chem., 1965, **4**, 763
16. K. Nakamoto, " Infrared and Raman Spectra of Inorganic and Coordination Compounds ", John Wiley, New York, 1963, p. 161
17. L. J. Bellamy, " The Infrared Spectra of Complex Molecules ", 3rd Ed, Methuen, London, 1966, p. 95
18. A. D. Liehr, J. Phys. Chem., 1967, **67**, 1314
19. R. L. Carlin, " Transition Metal Chemistry ", Ed, R. L. Carlin, Vol 1, Marcel Decker, New York, 1965.
20. A. B. P. Lever, " Inorganic Electronic Spectroscopy ", Elsevier, Amsterdam, 1984, p. 341
21. D. W. Meak, R. S. Drago and T. S. Piper, Inorg. Chem., 1962, **1**, 285
22. B. N. Figgis and J. Lewis, " Modern Coordination Chemistry ", Ed, T. Lewis and R. J. Wilkins, Interscience, New York, 1960.
23. B. N. Figgis and J . Lewis, Prog. Inorg. Chem., 1964, **6**, 87
24. Z. H. Chohan and F. Alam, Pak. J. Pharmacol., 1991, **8**, 8
25. Z. H. Chohan, Chem. Pharm. Bull., 1991, **39**, 1578
26. Z. H. Chohan and A. Rauf, J. Inorg. Biochem., 1994, **46**, 41

**Received: March 26, 1997 - Accepted: April 25, 1997 -**
**Received in revised camera-ready format: August 29, 1997**

